# *Thermococcus* sp. KS-1 PPIase as a fusion partner improving soluble production of aromatic amino acid decarboxylase

**DOI:** 10.1186/s13568-021-01340-3

**Published:** 2021-12-27

**Authors:** Takashi Koyanagi, Ayumi Hara, Kanako Kobayashi, Yuji Habara, Akira Nakagawa, Hiromichi Minami, Takane Katayama, Norihiko Misawa

**Affiliations:** 1 Department of Food Science, Ishikawa Prefectural University, Nonoichi, Ishikawa 921-8836 Japan; 2 Research Institute for Bioresources and Biotechnology, Ishikawa Prefectural University, Nonoichi, Ishikawa 921-8836 Japan; 3 Graduate School of Biostudies, Kyoto University, Sakyo-ku, 606-8501 Kyoto, Japan

**Keywords:** Peptidyl-prolyl *cis*-*trans* isomerase (PPIase), Molecular chaperon, *Thermococcus* sp. strain KS-1, Aromatic amino acid decarboxylase, *Escherichia coli*

## Abstract

Peptidyl-prolyl *cis*-*trans* isomerase (PPIase, EC 5.2.1.8) catalyzes the racemization reaction of proline residues on a polypeptide chain. This enzyme is also known to function as a molecular chaperon to stabilize protein conformation during the folding process. In this study, we noted FK506 binding protein (FKBP)-type PPIase from a hyperthemophilic archaeon *Thermococcus* sp. strain KS-1 (PPIase _KS−1_) to improve the solubility of *Pseudomonas putida* aromatic amino acid decarboxylase (AADC) that is an indispensable enzyme for fermentative production of plant isoquinoline alkaloids. AADC fused N-terminally with the PPIase _KS−1_ (PPIase _KS−1_-AADC), which was synthesized utilizing *Escherichia coli* host, showed improved solubility and, consequently, the cell-free extract from the recombinant strain exhibited 2.6- to 3.4-fold elevated AADC activity than that from the control strain that expressed the AADC gene without PPIase _KS−1_. On the other hand, its thermostability was slightly decreased by fusing PPIase _KS−1_. The recombinant *E. coli* cells expressing the PPIase _KS−1_-AADC gene produced dopamine and phenylethylamine from L-dopa and phenylalanine by two- and threefold faster, respectively, as compared with the control strain. We further demonstrated that the efficacy of PPIase _KS−1_-AADC in solubility and activity enhancement was a little but obviously higher than that of AADC fused N-terminally with NusA protein, which has been assumed to be the most effective protein solubilizer. These results suggest that PPIase _KS−1_ can be used as one of the best choices for producing heterologous proteins as active forms in *E. coli*.

## Key points

- Use of FKBP-type PPIase from *Thermococcus* sp. KS-1 as an effective molecular chaperon in *E. coli*.
- Solubility and activity enhancement in aromatic amino acid decarboxylase (AADC).
- Demonstration of the *Thermococcus* sp. KS-1 PPIase gene as a powerful fusion counterpart for the AADC gene.

## Introduction

Peptidyl-prolyl *cis*-*trans* isomerase (PPIase, EC 5.2.1.8) is an enzyme that catalyzes the racemization reaction of amino acid proline (Fischer and Schmid 1999). This protein is also known to act as a molecular chaperon to stabilize the folding process and structure of protein through the *cis*-*trans* isomerization of proline residues in a polypeptide chain. PPIase widely distributes in organisms belonging to all of three domains, i.e., bacteria, archaea, and eukaryotes including mammals and plants, thus constituting the large family responsible for the universal role of protein stabilization in living organisms. PPIase comprises three subfamilies, cyclophilin, FK506 binding protein (FKBP), and parvulin, and all of these have been shown to exhibit similar enzymatic activities (Fischer and Schmid 1999; Furutani 2000; Galat 2003; Göthel and Marahiel 1999; Lu et al. 2007; Maruyama et al. 2004; Shaw 2002; Tong and Jiang 2015).

Synthetic biology has empowered bacterial production of complex compounds with high commercial value. There are high demands for efficiently producing recombinant proteins with high solubility as well as desired protein activity. Indeed, solubility of proteins is one of the most important factors in microbial applications such as production of pharmaceutical proteins and small molecule compounds with enzymes. However, heterologous overexpression is in many cases troublesome because of the unmatched pair use of heterologous protein genes and host cells, leading to the formation of inclusion body containing a large amount of denatured target proteins. Thus, in case of *Escherichia coli*, various types of protein stabilizer have been developed, e.g., DnaK/DnaJ/GrpE and GroEL/ES (molecular chaperons co-expressed or fused with a target protein) (Bhandari and Houry 2009; Kyratsous et al. 2009), thioredoxin DsbA and DsbC (cysteine-bonds improvers fused to the N-terminus of a target protein) (Collins-Racie et al. 1995; Nozach et al. 2013), and N utilization substance A (NusA, high-performance protein solubilizer fused to the N-terminus of a target protein) (Davis et al. 1999). These systems can generally contribute to successful protein overproduction in the *E. coli* cells, but still have the probability that fails in the folding of soluble proteins case-dependently. It is thus desirable to retain the multiple options for functional expression systems as many as possible.

In this study, we employed the FKBP-type PPIase from a hyperthemophilic archaeon, *Thermococcus* sp. strain KS-1 (PPIase _KS−1_) (Furutani et al. 2000; Ideno et al. 2001, 2002, 2004; Iida et al. 1998; Misawa et al. 2011), as a molecular chaperon for attaining high-level production of *Pseudomonas putida* aromatic amino acid decarboxylase (AADC) in *E. coli* (Koyanagi et al. 2012). AADC catalyzes the reaction converting L-dopa into dopamine and requires pyridoxal phosphate for its enzymatic activity. Dopamine is an important neurotransmitter and is often used as a supplement for maintaining human body condition, thus, the effective expression of the AADC gene would benefit microbial production of this compound. Furthermore, the AADC reaction constitutes an important step for our recently developed plant isoquinoline alkaloid production system using engineered *E. coli*, since the isoquinoline backbone is built by the condensation of aromatic amine and aromatic aldehyde. Isoquinoline alkaloids have been known as pharmaceutically important compounds, therefore attaining high activity of a key enzyme AADC in isoquinoline alkaloid synthesis pathway inside the *E. coli* cells should directly be linked with future increased industrial production level of these useful compounds. We here demonstrated an archaeal FKBP-type PPIase _KS−1_ as a powerful fusion counterpart for the heterologous functional expression of the AADC gene in *E. coli* as the host cell.

## Methods

### Bacterial strains and plasmids

**Fig. 1:**
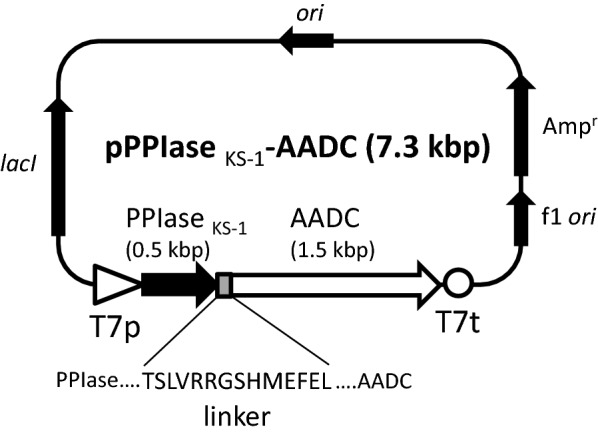
Construction of the expression vector for PPIase _KS−1_-AADC (pPPIase _KS−1_-AADC). The structural gene of PPIase _KS−1_-AADC was expressed under the control of the T7 promoter (T7p) and the T7 terminator (T7t). The 14 amino acids linker was indicated between PPIase _KS−1_ and AADC

*E. coli* DH5α was generally used as a host for genetic manipulations, and *E. coli* BL21(DE3) was used as a host for protein overproduction. The plasmids pET-3a and pET-43.1a (Merck, Darmstadt, Germany) were used as cloning and expression vectors for the AADC and NusA-AADC structural genes respectively. The structural gene of AADC (*aadc*, GenBank accession no. BK006920.1) was amplified from the genomic DNA of *P. putida* KT2440 by the polymerase chain reaction (PCR) using PrimeStar GXL DNA polymerase (TakaraBio, Shiga, Japan) with a primer pair 5′-AAACCCCATATGACCCCCGAACAATTCCG-3′ and 5′-AAAGGATCCTCAGCCCTTGATCACGTCCTG-3′ (The *Nde*I and *Bam*HI restriction sites are underlined respectively). The amplified fragments (approximately 1.5 kbp) were treated with *Nde*I and *Bam*HI and inserted into pET-3a digested by the same restriction enzymes. The 1.2 kbp fragment containing the *lacI* gene, amplified by PCR using pET-43.1a as a template and a primer pair 5′-GGCGCCATCTCCTTGGATCCCGGACACCATCGAAT-3′ and 5′-CCGCAAGGAATGGTGCTAGTCATGCCCCGCGCCCA-3′, was inserted into *Sph*I site of the resulting plasmid by using In-Fusion® HD Cloning Kit (15 bp homologous nucleotides in primers used for recombination are underlined). The constructed plasmid (pAADC) was used for overproduction of wild-type *P. putida* AADC. The *Thermococcus* sp. KS-1 FKBP-type PPIase gene (GenBank accession no. AB012209.1) was fused to N-terminus of AADC by inserting the *aadc* gene into pFusion-F87V (Misawa et al. 2011). pFusion-F87V was derived from pET-21d (Merck), therefore PPIase _KS− 1_-AADC could share the same T7 expression system and ColE1 origin of replication with wild-type AADC described above. The *aadc* gene was PCR-amplified by using *P. putida* KT2440 genomic DNA as a template and a primer pair 5′-CCCGAGCTCATGACCCCCGAACAATTCCG-3′ and 5′-CCCCTCGAGTCAGCCCTTGATCACGTCCTG-3′ (The *Sac*I and *Xho*I restriction sites are underlined respectively). After digestion with *Sac*I and *Xho*I, the fragment was inserted into the similarly-cut pFusion-F87V to construct pPPIase _KS− 1_-AADC. The NusA-AADC-expressing plasmid (pNusA-AADC) was constructed by amplifying the *aadc* gene from pPPIase _KS− 1_-AADC by using a primer pair 5′-GCTACTAGTCTGGTTCCGCG-3′ and 5′-CCCCTCGAGTCAGCCCTTGATCACGTCCTG-3′ (The *Spe*I and *Xho*I restriction sites are underlined respectively), and the amplified fragments were inserted into pET-43.1a at the *Spe*I and *Xho*I sites. Summary of the expression construct of pPPIase _KS− 1_-AADC was shown in Fig. 1. All PCR-amplified regions were sequenced to confirm no introduction of nucleotide errors.

## Media and chemicals

Luria-Bertani (LB) (Difco) was routinely used for cultivation of the *E. coli* strains. When the production of dopamine and phenylethylamine was performed, M9-0.2 (w/v) % glucose minimal medium supplemented with 1 mM L-dopa or L-phenylalanine were used for cultivation. Ampicillin was added to media at 100 µg/ml to maintain the expression vectors in the *E. coli* cells. L-Dopa, dopamine, L-phenylalanine, and phenylethylamine were purchased from NakaraiTesque Co. (Kyoto, Japan).

### Overproduction of AADC, NusA-AADC, and PPIase _KS−1_-AADC

The *E. coli* BL21(DE3) derivatives carrying pAADC, pNusA-AADC, or pPPIase _KS−1_-AADC were cultivated in 50 mL LB at 25 °C with shaking at 140 rpm, and when the turbidity at 600 nm reached to 0.4−0.6, isopropyl-β-d-thiogalactopyranoside (IPTG) was added at the final concentration of 0.2 mM. Temperature was then changed to 18 °C, and the cultivation was continued with shaking for 18 h. The cells were harvested by centrifugation at 5000×*g* for 8 min, and were disrupted by ultrasonication in 50 mM potassium phosphate (pH 7.0) containing 4 mM 2-mercaptoethanol (2-ME) and 200 µM pyridoxal 5′-phosphate (PLP). Cell-free extract and insoluble cellular debris were separated by following centrifugation at 15,000×*g* for 10 min. The solubility of the proteins were analyzed with SDS-PAGE by applying cell-free extracts and cellular debris equivalent to 10 µg of the cells.

### AADC activity assay

Cell-free extracts were evaluated for the AADC activity by using the methods we described in the previous study with some modifications (Koyanagi et al. 2012). The reaction solution comprised 50 mM potassium phosphate (pH 8.0), 2 mM 2-ME, 50 µM PLP and 1 mM L-dopa. One milliliter of this reaction mixture was preincubated for 5 min at 30 °C, followed by initiation of the reaction by adding 20 µL of the cell-free extract, and stopping the reaction by adding HCl at the final concentration of 0.1 M. The reaction products were analyzed by HPLC equipped with a Discovery HS F5 column (Supelco, St. Louis, MO). The elution was performed at a flow rate of 0.5 mL/min by increasing the concentration of acetonitrile from 3 to 20% in 10 mM ammonium formate buffer (pH 3.0), and the detection of L-dopa and dopamine was performed by measuring the absorbance at 280 nm.

### Production of dopamine and phenylethylamine by recombinant *E. coli* strains

The AADC- and PPIase _KS− 1_-AADC-expressing *E. coli* cells were evaluated for their dopamine and phenylethylamine production ability by adding 1 mM L-dopa and L-phenylalanine to medium as substrates, respectively. The cells were cultivated in 50 mL M9 minimal medium at 25 °C with shaking at 140 rpm, and IPTG was added at the concentration of 0.1 mM when the turbidity at 600 nm reached to 0.4−0.6. The samples of 1 mL were withdrawn at the times indicated and centrifuged for 1 min at 15,000×*g*, and the concentrations of dopamine and phenylethylamine were measured by HPLC. Detection of L-dopa and dopamine was performed as described above, whereas L-phenylalanine and phenylethylamine were fluorescent-labeled by AccQ-tag (contained in AccQ-Fluor™ Reagent Kit, Waters, Milford, MA) prior to the HPLC analysis. AccQ-tag amino acid analysis column (Waters) was used to separate the compounds, and fluorescence was detected with excitation wavelength at 250 nm and emission at 395 nm. Elution program was set according to the manufacturer’s instruction.

## Results

### Overproduction of AADC and comparison of solubility among the expression systems

**Fig. 2:**
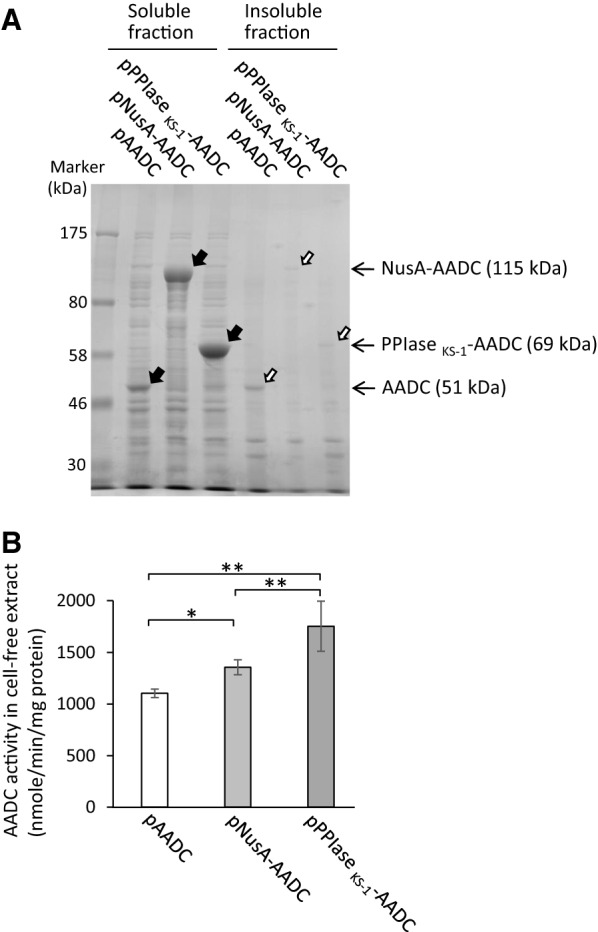
Overproduction of AADC, PPIase _KS−1_-AADC and NusA-AADC. **A** Cell-free extract from each *E.coli* overexpressing strain was analyzed by SDS-PAGE. Prestained Protein Marker, Broad Range (New England Biolabs) was used as a molecular weight marker. Soluble and insoluble overexpressed target proteins were indicated by filled and open arrows respectively. **B** AADC activity in cell-free extracts of the respective overexpressing strains. The values are indicated as mean ± standard deviation. Statistical significance (*p < 0.05; **p < 0.01) was determined with Tukey’s multiple comparisons test for four measurements

*E. coli* BL21(DE3) carrying pAADC, pNusA-AADC, or pPPIase _KS−1_-AADC were cultivated in LB and the expression of target genes from the T7 promoter were induced by IPTG. In the PPIase _KS−1_-AADC fusion construct, a 14 amino acids linker (TSLVRRGSHMEFEL) was introduced between two structural genes of the proteins, since this length had been found to be effective for stabilizing the *Bacillus megaterium* P450 BM3 (variant F87V) when expressed in *E. coli* BL21(DE3) (Misawa et al. 2011). The cell-free extracts and the cell debris were analyzed for their protein contents by SDS-PAGE (Fig. 2A). The soluble form of wild-type AADC was detectable, but the significant amount was seen in the insoluble fraction (pAADC). On the other hand, NusA-AADC was highly soluble as compared to the wild-type protein and only a little amount appeared as insoluble form (pNusA-AADC). PPIase _KS−1_-AADC was also produced almost in the soluble fraction, and the amount of the protein detected in the insoluble fraction was similar to the case of NusA-AADC (pPPIase _KS−1_-AADC). These results indicated that PPIase _KS−1_ greatly improved the solubility of AADC, and the effectiveness of PPIase _KS−1_ was comparable to the NusA system, which is regarded as one of the most efficient protein solubilizer generally used among the *E. coli* overexpression constructs.

The AADC activity was measured using these cell-free extracts, and the results were shown in Fig. 2B. The activities for NusA-AADC and PPIase _KS−1_-AADC were significantly higher than AADC without fusion partner, well reflecting the high solubility of the NusA and PPIase _KS−1_ systems. Although the thickness of protein bands were almost similar between NusA- and PPIase _KS−1_-fusions, the AADC activity toward L-dopa was significantly higher in PPIase _KS−1_-AADC as compared to NusA-AADC (1750 ± 240 vs. 1360 ± 70 nmole/min/mg protein respectively), thus indicating that the efficiency of the PPIase _KS−1_ system is comparable or even superior to NusA.

### Thermostability of PPIase _KS−1_-fused AADC

**Fig. 3:**
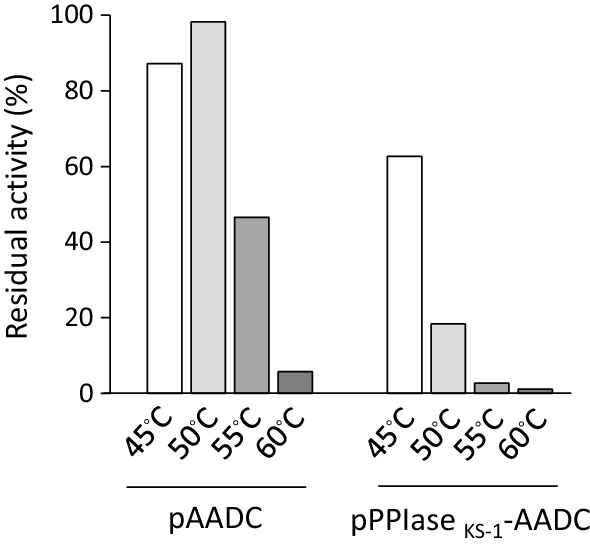
Thermostability of wild-type AADC and PPIase _KS−1_-AADC. Cell-free extracts of wild-type AADC- and PPIase _KS−1_-AADC-producing strains were incubated at 45, 50, 55 and 60 °C for 30 min, and the residual enzymatic activity was measured

In the previous study, thermotolerance of the *B. megaterium* P450 BM3 (variant F87V) protein was improved by the existence of PPIase _KS−1_ at N-terminus (Misawa et al. 2011). Based on this result, PPIase _KS−1_-fused AADC was tested for its thermostability by incubating the cell-free extracts prepared from the *E. coli* strain expressing the wild-type or PPIase _KS−1_-fused AADC gene (Fig. 3). PPIase _KS−1_-fused P450 BM3 heterologously produced in the *E. coli* cells restored the enzymatic activity at 43.9% upon 20-min heat treatment of cell-free extract at 60ºC, while the recombinant protein without PPIase _KS−1_-fusion recorded not more than 27.7%. PPIase _KS−1_-AADC, however, was found to be weaker than non-fused AADC against heat in this study, as shown in Fig. 3. We heat-treated the cell-free extract from 45 to 60 °C for 30 min, but the residual activity of PPIase _KS−1_-AADC was clearly inferior to that of AADC. This result indicates that thermostability of PPIase _KS−1_-fusion is protein-species dependent.

### Dopamine and phenylethylamine production by the PPIase _KS−1_-AADC overproducing strain

**Fig. 4:**
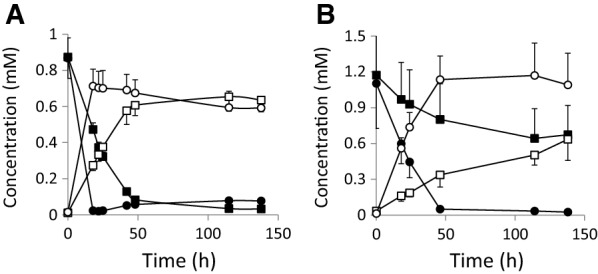
Dopamine and phenylethylamine production by wild-type AADC (squares) and PPIase _KS−1_-AADC (circles) overproducing strains. **A** Production of dopamine (open symbols) from L-dopa (closed symbols) and **B** production of phenylethylamine (open symbols) from L-phenylalanine (closed symbols) are shown. The experiments were repeated three times for (**A**) and twice for (**B**), and values were indicated as mean ± standard deviation

Next, we evaluated the production ability of aromatic amines (dopamine and phenylethylamine) of the strains expressing the wild-type AADC and PPIase _KS−1_-AADC genes. AADC is capable of converting L-phenylalanine into phenylethylamine, though the reaction is weaker than converting L-dopa to dopamine (Koyanagi et al. 2012). M9 minimal media containing 1 mM L-dopa or L-phenylalanine were used in this experiment, since L-dopa and dopamine are unstable in the complete medium LB and easily converted into black melanin-like pigment during cultivation. Figure 4A, B show the conversion of L-dopa and L-phenylalanine into dopamine and phenylethylamine, respectively. Wild-type AADC-producing *E. coli* strain exhibited the dopamine production achieving the final concentration of 0.6 mM at 42 h after the start of cultivation, but PPIase _KS−1_-AADC-expressing strain produced higher amount of 0.7 mM at earlier time point around 18 h (Fig. 4A). At this time point, the accumulation level of dopamine was approximately 2.6-fold higher for PPIase _KS−1_-AADC-producing strain than wild-type AADC-producing strain. Phenylethylamine accumulation was also higher in PPIase _KS−1_-AADC-producing strain (1.1 mM at 46 h cultivation) than that of wild-type AADC-producing strain (0.3 mM at the same time point, 3.4-fold lower than PPIase _KS−1_-AADC). The latter strain was capable of producing phenylethylamine not more than 0.6 mM even after 138 h from the start of cultivation. PPIase _KS−1_-AADC fusion thus elevated the production level of active AADC molecules, leading to the increment of dopamine and phenylethylamine accumulation.

## Discussion

In this study, we obtained results that the solubility of *P. putida*-derived AADC linked to FKBP-type PPIase _KS−1_ at the N-terminus was significantly increased in recombinant *E. coli* cells, as compared with the non-fused protein. A similar result has been shown in P450 BM3 (variant F87V) from *Bacillus megaterium* (Misawa et al. 2011), indicating that function of FKBP-type PPIase _KS−1_ as a chaperon is useful for the production of soluble proteins with active forms. In addition, PPIase _KS−1_ showed comparable solubilizing efficiency with NusA, which is involved in the heterologous expression system of *E. coli* with the highest solubilizing capacity so far reported. Another FKBP-type PPIase of *E. coli* origin, SlyD, has been evaluated as a N-terminal fusion counterpart of various aggregation-prone heterologous proteins including *Candida antarctica* lipase B (CalB), and its high solubilizing activity was confirmed in *E. coli* (Han et al. 2007; Seo et al. 2009). Geitner et al. (2013) expressed the gene encoding an active parvulin domain (Par2) of the *E. coli* periplasmic prolyl isomerase SurA as a chimeric protein with chaperone domain of SlyD, and found that the folding activity was dramatically increased to 1500-fold higher than the wild-type SurA. SlyD and PPIase _KS−1_ are both classified into the FKBP-C superfamily protein, but both are derived from distinct domains, i.e., bacteria and archaea, respectively, and shared only 30% identity in their amino acid sequences. Despite these significant differences, both proteins seem to act as similarly effective folding enhancer when used as the N-terminal fusion counterpart in *E. coli*.

It was found that the thermal stability of AADC was slightly weakened by fusing with PPIase _KS−1_, although elevated thermal stability was observed in case of using P450 BM3 (variant F87V) instead of AADC (Misawa e al. 2011). The reason for the unfavorable result is unknown at the present, but, since the structure of the protein becomes bulkier than the wild-type AADC, by fusing PPIase _KS−1_, it is possible that denaturation or decomposition of the PPIase _KS−1_ and AADC fusion protein were promoted due to intense structural change provoked by heat treatment. This result is also conflicting with improvement of protein solubility and activity, and future follow-up survey is required.

However, *Thermococcus* sp. KS-1 PPIase is sufficiently small, only 17.5 kDa, when compared to NusA that is a large protein of 54.9 kDa, so that the use of such a small PPIase _KS−1_ protein as a fusion counterpart is obviously advantageous and the size of a plasmid constructed can be reduced. In recent synthetic biology, it is necessary to simultaneously express a large number of heterologous genes in the same cell, therefore the compactness of PPIase _KS−1_ should surely be useful. *P. putida* AADC is a protein used in the synthetic-biological production of plant isoquinoline alkaloids using *E. coli* cells (Kim et al. 2013; Matsumura et al. 2017; Minami et al. 2008; Nakagawa et al. 2011, 2012, 2014, 2016). Since L-dopa is unstable in production media (Nakagawa et al. 2011), rapid conversion from L-dopa to dopamine seems to be an important reaction step for effectively forming the isoquinoline skeleton. Thus, improved solubility of this enzyme may further contribute not only to microbial production of bioactive compound dopamine, but also to future industrial production of pharmaceutically important isoquinoline alkaloids. Thus, *Thermococcus* sp. KS-1 PPIase can be a good choice for improving the production property of heterologous proteins, which contribute to an important issue in synthetic biology to efficiently provide starting materials or intermediates.

## Data Availability

The datasets generated during and/or analysed during the current study are available from the corresponding author on reasonable request.
